# Modeling and Measuring Signal Relay in Noisy Directed Migration of Cell Groups

**DOI:** 10.1371/journal.pcbi.1003041

**Published:** 2013-05-02

**Authors:** Can Guven, Erin Rericha, Edward Ott, Wolfgang Losert

**Affiliations:** 1 Department of Physics, University of Maryland, College Park, Maryland, United States of America; 2 Institute for Research in Electronics and Applied Physics, University of Maryland, College Park, Maryland, United States of America; 3 Department of Physics and Astronomy, Vanderbilt University, Nashville, Tennessee, United States of America; 4 Department of Electrical and Computer Engineering, University of Maryland, College Park, Maryland, United States of America; University of Virginia, United States of America

## Abstract

We develop a coarse-grained stochastic model for the influence of signal relay on the collective behavior of migrating *Dictyostelium discoideum* cells. In the experiment, cells display a range of collective migration patterns, including uncorrelated motion, formation of partially localized streams, and clumping, depending on the type of cell and the strength of the external, linear concentration gradient of the signaling molecule cyclic adenosine monophosphate (cAMP). From our model, we find that the pattern of migration can be quantitatively described by the competition of two processes, the secretion rate of cAMP by the cells and the degradation rate of cAMP in the gradient chamber. Model simulations are compared to experiments for a wide range of strengths of an external linear-gradient signal. With degradation, the model secreting cells form streams and efficiently transverse the gradient, but without degradation, we find that model secreting cells form clumps without streaming. This indicates that the observed effective collective migration in streams requires not only signal relay but also degradation of the signal. In addition, our model allows us to detect and quantify precursors of correlated motion, even when cells do not exhibit obvious streaming.

## Author Summary

Collective cell migration is observed in various biological processes including angiogenesis, gastrulation, fruiting body formation, and wound healing. *Dictyostelium discoideum*, for example, exhibits highly dynamic patterns such as streams and clumps during its early phases of collective motion and has served as a model organism for the study of collective migration. In this study, facilitated by experiments, we develop a conceptual, minimalistic, computational model to analyze the dynamical processes leading to the emergence of collective patterns and the associated dependence on the external injection of a cAMP signal, the intercellular cAMP secretion rate, and the cAMP degradation rate. We demonstrate that degradation is necessary to reproduce the experimentally observed collective migration patterns, and show how our model can be utilized to uncover basic dependences of migration modes on cell characteristics. Our numerical observations elucidate the different possible types of motion and quantify the onset of collective motion. Thus, the model allows us to distinguish noisy motion guided by the external signal from weakly correlated motion.

## Introduction

Eukaryotic cells frequently transduce external chemical gradients into directed cell migration [1], a phenomenon known as chemotaxis. Seminal work in the last few decades has identified components of the intracellular biochemical networks mediating cell response to external chemical gradients and found that responsive components such as the phosphoinositide lipids (PIPs), PI3K, and PTEN are highly conserved across cell types. In these efforts, our model organism (*Dictyostelium discoideum*) has been a useful source for discovery of network components and the development of quantitative models exploring plausible mechanisms for mediating directional sensing. Despite the vast similarities in gradient detection among *D. discoideum* and mammalian cells including neutrophils and neurons, *D. discoideum* chemotaxis displays a striking collective phenomenon not often found in other cell types where *D. discoideum* cells responding to the extracellular chemical signal cyclic-AMP (cAMP) tend to migrate in a head-to-tail fashion termed streams. In response to an external cAMP cue, *D. discoideum* cells synthesize and secrete cAMP relaying the initial signal to nearby cells. Many cell types, including neutrophils, macrophages, and epithelial cells, have potential signal relay loops, but they do not tend to migrate in streams in a standard chemotaxis assay.

Building on previous work [2]–[5], we develop a minimalistic model for *D. discoideum* migration and signal relay in a linear gradient. Our model incorporates recent experimental measurements on cell migration persistence [6], independence of signal strength [5], and migration mechanism and lag in reorienting to signals [7]. We use the model to ask what aspects of the signal relay loop promote streaming. We find that a balance between fast secretion and degradation are needed to match experimental observations. To constrain the migration parameters, we measure the time autocorrelations and the fluctuations of the cell motion from our experimental systems and we suggest the possible use of these metrics to find evidence of signal relay in cells that do not display streams. Our efforts are motivated by recent experiments on *D. discoideum*, that show a notable visual distinction between cells that relay signals, and cells that both relay and degrade the signal. Wild-type cells, which emit cAMP and degrade cAMP, can form streams where cells are aligned head to tail, while mutant PDE1- cells that are unable to degrade cAMP form transient, aberrant streams that lead to clusters [8].

When food is plentiful, *D. discoideum* cells exist as single cells and chemotax towards the bacterial metabolic product folic acid. When food is removed, *D. discoideum* transitions from single cell to collective behavior - through the spontaneous secretion and detection of cAMP. The cooperative behavior of this spontaneous transition was found to follow Winfield synchronization and, the emergence of pulsatile, signaling centers is beautifully described in [5]. These pulses travel through a population of *D. discoideum* in spiral waves [9], [10]. Secretion of the extracellular phosphodiesterase (PDE1) is essential for the spontaneous transition [11]. Each pulse of external cAMP detected by cells results in an increase in gene expression promoting collective behavior [12], and after 4–6 hours of cAMP mediated development, cells begin to aggregate. In order to determine the essentials for chemotaxis and streaming separate from those needed for development, researchers often provide exogenous pulses of cAMP [12], [13]. From these studies, it has been found that cAMP secretion is essential for streaming, but not for chemotaxis. Cells lacking adenyl cyclase A, the enzyme primarily responsible for internal cAMP production during aggregation, will chemotax to cAMP without forming streams [14]. Development and chemotaxis to cAMP in cells lacking the gene for PDE1 can be rescued through periodic addition of partially purified PDE1. Cells lacking PDE1 secretion will chemotax to cAMP and form transient streams to a central source of cAMP, though in linear gradients, such as the under agar assays, the streams appear thicker than wild type [8]. Spontaneous aggregation by developed PDE1 null cells can be recovered with the addition of a uniform bolus of exogenous PDE1, though the bolus is insufficient to recover the spatial extent of the streams. Because we intend to examine a minimalistic model, we include continuous, local cAMP secretion and a constant background of cAMP degradation.

The dynamics of the pre-aggregation stage of *D. discoideum* development was analyzed by Potel and Mackay [15], where they observed the motion of cells and calculated various dynamic quantities, such as the mean speed and the mean square displacement of cells and used Furth's persistent motion model [16], [17] to explain their observations. Futrelle et al. [18] investigated chemotactic response to an external signal for early, middle and late developed cells for different duration and frequencies of cAMP pulses. In particular, the chemotactic index and the speed of the cells during development were analyzed, and significant timescales that define the dynamics were extracted, including the response time to a change in cAMP gradient which they estimated to be on the order of 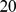 seconds. Gingle [19] measured the smallest cell density 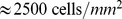, above which collective motion occurs. Gingle and Robertson [20] showed that this limit density depends on the development time of the cells.

The spontaneous emergence of traveling waves in a population of *D. discoideum* cells has attracted interest of the mathematics and physics communities and lead to the development of several computational models to test hypothesis for mechanisms involving signal transduction, signal relay, and gradient sensing. Pioneering work by Martiel and Goldbeter used a differential equation approach based on the receptor activation and desensitization dynamics [21] to explain the pulses of cyclic AMP. In addition to modeling the receptor dynamics, following models studied mechanisms in *D. discoideum* chemotaxis including wave propagation of cAMP signals in an inhomogeneous excitable medium [9], [22]–[25], directional sensing via receptor activation followed by further intracellular signaling [26]–[28], and physical forces that regulate cell-cell or cell-surface interactions [29]–[32].

Other models of chemotaxis focus on stochastic aspects of the cellular processes. These models discuss mechanisms including stochastic dynamics of directional sensing and speed control [2], [33]–[36], ″memory″ associated with membrane deformations [37]–[39], extension of new pseudopods conditional on the locations of existing ones [40]–[41]. Recent models of chemotaxis study the effects of noise due to fluctuations in receptor binding as well as the noise arising from subsequent internal responses [4], [42]–[46]. In the simplest models directional sensing is represented as stochastic dynamics of a single angular variable (which represents the density asymmetry of both the occupied receptors and further downstream processes such as 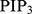 regulation). Schienbein et al. [33] showed that the dynamics of the stochastic angle agrees very well with the directional sensing dynamics of granulocytes during galvanotaxis. The stochastic angle model was also implemented for *D. discoideum* chemotaxis by including receptor kinetics and chemical gradient steepness [4]. In this work we choose to capture the stochastic effects by associating the stochasticity of the previously described angular variable with the measured fluctuations in the direction of motion.

The focus of our study is on modeling, simulating, and analyzing collective motion arising from chemotaxis and signal relay. While collective motion, chemotaxis, and signal relay have all been investigated before, this work focuses on collective behavior in the presence of a linear gradient without fluid flow. The linear, no-flow gradient geometry has been used in conjunction with Zigmond chambers and underagar assays but was cumbersome and often replaced with point sources, such as a micropipette, which leads to convergent cell trajectories even in the absence of signal relay. A linear gradient has been recently incorporated into a microfluidic system which can simultaneously monitor multiple gradient conditions and cell lines (using EZ-TAXIScan system (ECI, Japan) [47]). By monitoring many parallel conditions we are able to clearly analyze signal relay and differentiate different types of collective motion. It also allows us to validate metrics for detection of collective behavior that should be useful for the analysis of a number of other investigations of cell signaling that are starting to be carried out in this signal geometry. Linear gradients have been introduced for quantitative studies of gradient sensing, but recent work in microfluidics devices has been carried out in chambers with fluid flow which flushes out signal relay (e.g., in Refs. [45], [46]).

The controlled linear gradient allows us develop a quantitative phenotype for the onset of signal relay between cells. We are able to tune the relative strength of signal relay continuously, by varying the linear gradient strength. This allows us to measure collective behavior based on correlations between cell trajectories. We anticipate that our systematic studies will be valuable for a broad range of investigations of collective cell behavior. Indeed cell trajectories in such linear gradient chambers are starting to be collected to investigate signaling pathways that regulate chemotaxis in various types of cells (e.g., *D. discoideum* [48], neutrophils [49], [50], eosinophils [51], and osteoclasts [52]).

## Results

### Experiments in linear chemical gradient classifies the collective response of relay systems to externally imposed signals

**Figure 1:**
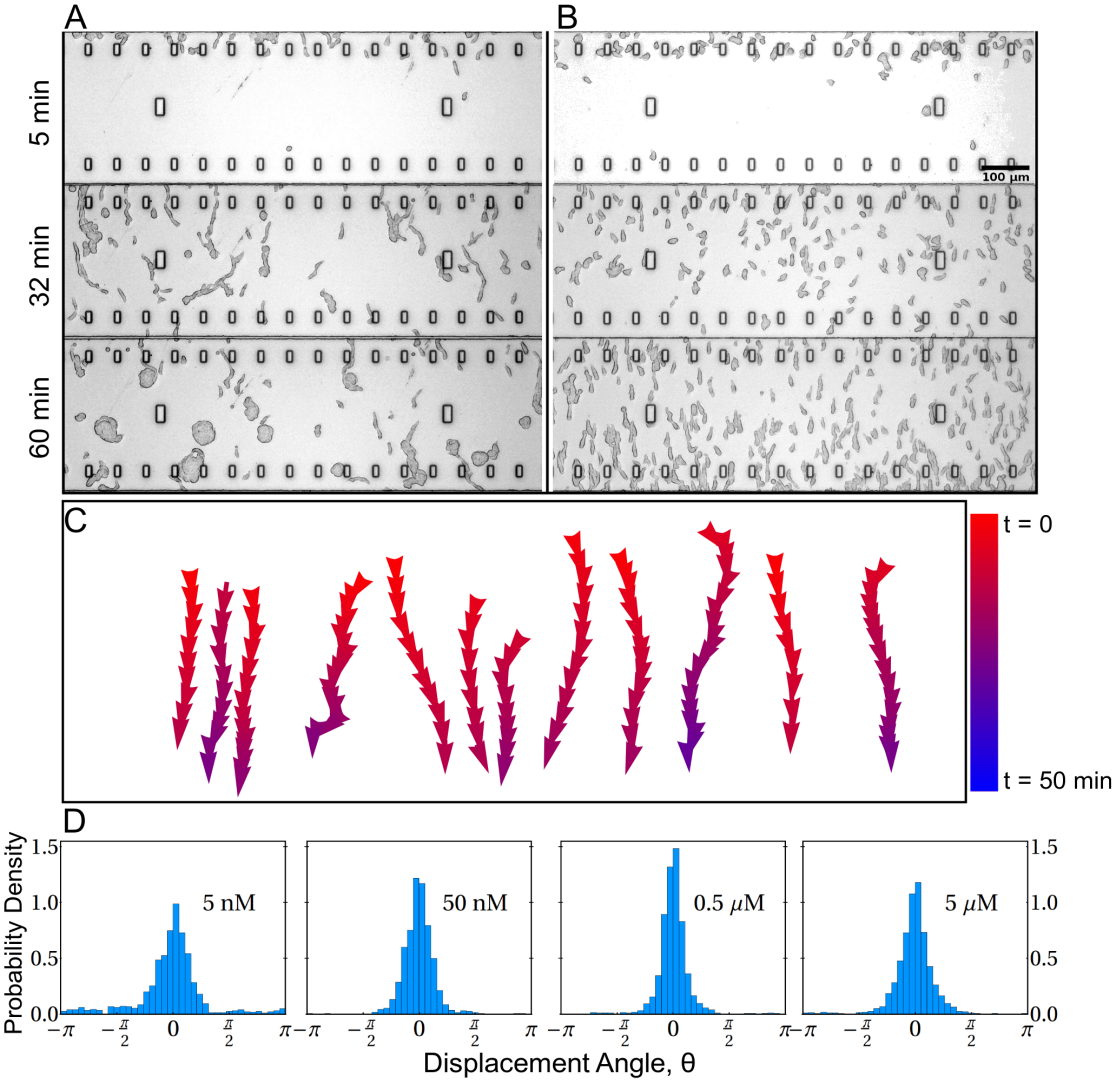
Time lapse images during the chemotaxis of wild-type and mutant cells in linear cAMP gradient. (A) Wild-type cells can relay the signal by secreting cAMP from their tails. They form streams which are unstable towards swirling clumps. (B) The mutant cells (aca-) lacking the ACA enzyme cannot secrete cAMP and thus undergo uniform motion in the direction of the external cAMP gradient. (C) Some representative tracks of aca- cells obtained with the tracking algorithm. Vector displacements along the tracks are color coded according to real time. (D) Distributions of the angle representing the displacement of cells exposed to different constant gradient amplitudes with respect to the vertical axis. The panel labels (5 nM to 5 µ*M*) denote the cAMP concentration in the reservoir.

The EZ-TAXIScan system uses an etched silicon chip to form 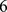 separate channels for chemotaxis experiments in a linear geometry [47]. Each channel contains two buffer wells on opposite sides of a thin, terraced gap (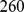 microns long, 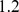 mm wide and 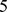 microns deep). Cells are gently pipetted into one well and allowed to settle to the glass surface. The opposite channel is filled with cAMP and diffusion sets a linear gradient in the channel within 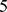 minutes. Cells, responding to the external signal enter the terraced region and travel 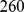 microns towards the other side. Parallel to the edges of the terrace are small columns (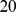 microns long, 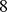 microns apart) that set the vertical spacing, but provide little impedance to cell motion. If not modulated by cAMP or by PDE1 secreted by the cells, the imposed gradient stays constant at least for 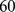 minutes [47], [51]. This type of setup provides a good signaling geometry for separating the effect of intercellular communication and an imposed gradient. Fig. 1A and Fig. 1B show time lapse images of wild-type cells and mutant cells under the influence of a linear (downward in the figures) cAMP gradient. At 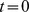 cells placed in a reservoir without cAMP begin to move into the chamber (at the top boundary in the figures). Although the cells are initially introduced uniformly in the horizontal direction (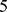 min panel of Fig. 1A and Fig. 1B), wild-type cells are attracted to each other and form streams (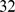 min panel of Fig. 1A), which in this example evolve to swirling groups (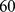 min. panel of Fig. 1A). The mutual attraction of the cells is due to the enzyme adenyl cyclase A (ACA) localized at the back of the cells [14]. ACA synthesizes intracellular cAMP, which diffuses into the extracellular medium. As shown in Fig. 1B, mutant cells (aca-), lacking ACA, do not exhibit collective motion and, throughout the time-course of the experiment, move without streaming or clumping in the direction of the external cAMP gradient.

To analyze these observed migratory behaviors, we use a cell tracking algorithm to determine cell displacement vectors over a short time interval 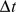 of the position of the center of the imaged intensity of each cell. We define a motion angle 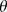 as the angle of a cell's displacement vector with respect to the imposed cAMP gradient. Fig. 1C shows representative tracks of cells during chemotaxis (color coded according to real time). Fig. 1D shows the distributions of the angle 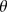 for aca- cells, subject to four different external cAMP gradient strengths, increasing by a factor of 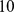 from panel to panel. The spread of 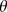 reflects the competition between noise and the ability of cells to sense and react to the gradient. Note that the width of the distributions first decreases with increasing gradient strength then decreases indicating an optimum. This finding agrees with observations of Fuller et al. [45], which shows that the chemotactic response is limited by external noise (noise due to receptor-ligand binding) for small local cAMP concentration and by internal noise (noise due to subsequent internal signaling) for higher local cAMP concentration.

The distributions in Fig. 1D show that the cells do not always orient in the direction of the extracellular gradient 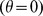. As discussed in [53] the gradient-sensing mechanism is stochastic with many sources of noise that can cause random deviation from the direction of the external gradient. Our data for the angular distributions suggest that above a threshold gradient the cell orientation is independent of the gradient strength. Below this threshold (e.g., see the 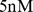 panel of Fig. 1D), the width of the 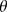 distribution increases with decrease of the gradient [45]. In what follows we focus on the regime where the cell migration is less sensitive to the gradient strength.

**Figure 2:**
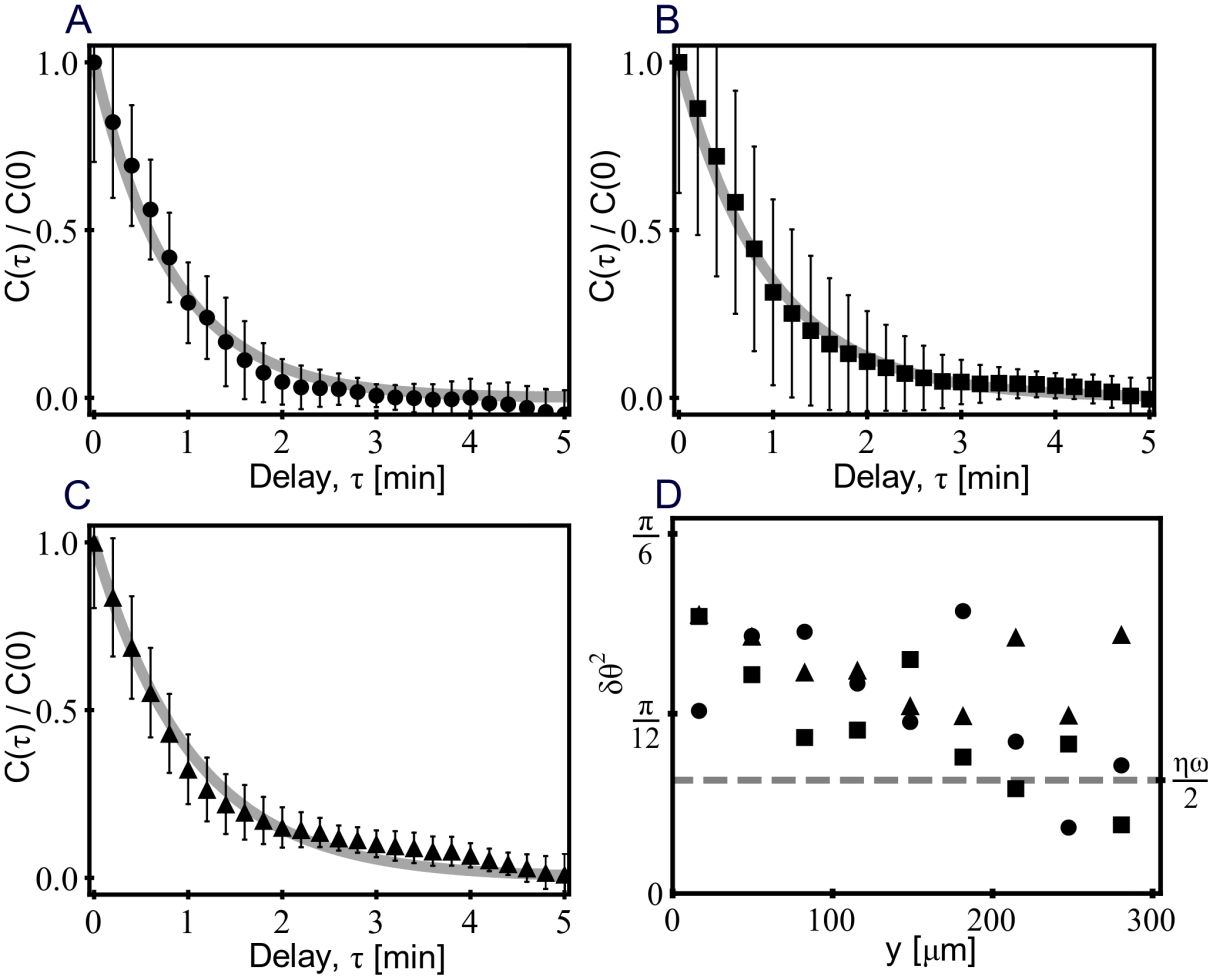
The time autocorrelation and variance of *θ*. 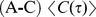
 versus τ for three different imposed cAMP gradient strengths corresponding to cAMP concentrations of 50 *nM* (black bullet), 0.5 µ*M* (black square) and 5 µ*M* (black triangle) in the reservoir on the cell exit side of the gradient chamber. The solid lines are best fits to 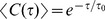 yielding values for 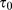 of 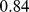 min, 0.94 min and 1 min. Autocorrelations are obtained from 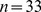, 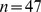, and 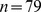 cells, respectively. Error bars represent the standard deviation. (D) The variance 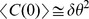, versus the distance 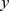 from the cell input side of the gradient chamber for the three gradient strengths in Figs. 2A–C is plotted using the same symbols black bullet, black square and black triangle.

For several representative cells, Figs. 2A–C show the time autocorrelation of 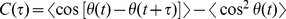, where the angle brackets denote an average over time for cells that are located in the region between the cell exit plane and the mid plane of the gradient chamber (lower half of the panels in Figs. 1A and B, (the number of cells are 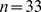, 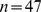, and 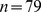, respectively)). The reason for restricting the averaging to the half of the chamber on the cell exit side is to eliminate any bias of the cell orientation angle distribution due to influence of the process of entry into the chamber. For small angles (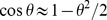) the autocorrelation is 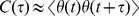. The variance of 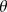, 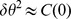, is plotted as a function of the distance from the starting point of the cells in Fig. 2D for the three different gradient strengths. In the next section we develop a model which estimates the level of the fluctuations in the displacement (dashed line in Fig. 2D). Previous studies on eukaryotic *HaCaT* cells highlight the dependence of velocity autocorrelations on two time scales [37]. Nevertheless, we see from Figs. 2A–C that 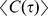 can be well fitted to a dependence of the form 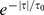 parametrized by the single characteristic time 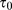. The fits for the average correlations 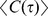 for the individual gradient strengths are displayed in Figs. 2A–C. The single time scale, 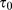, is approximately constant over the two orders of magnitude in the external cAMP gradient strengths (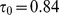 min, 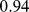 min and 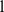 min for Fig. 2A, Fig. 2B, and Fig. 2C). This time scale is roughly consistent with the dynamics of contractions of cells [31].

### Modeling collective migration of *D. discoideum* in a linear gradient chamber enables quantitative description of collective responses to externally imposed signals

The characteristic size of eukaryotic cells is an order of magnitude larger than that of bacterial cells, and, in contrast with the sensing by bacterial cells, eukaryotic cells can sense the difference in chemoattractant concentration between the front and the back of a cell, thus detecting spatial gradients without moving. For *D. discoideum*, gradient sensing is accomplished via a G-protein coupled receptor and downstream signaling pathways [54]. Models of chemotaxis treating the cAMP signal transduction mechanism, including the biochemical details such as receptor desensitization [21] and adaptation [55], demonstrate the emergence of the experimentally observed cAMP waves. In the present paper our modeling approach will differ somewhat from past works (e.g., Refs. [9], [22], [24], [56]) in that we seek a model that is simple enough that its relatively few parameters can be inferred from experiments, yet is still capable of capturing the distinctions between streams and clumps seen in our experiments on *D. discoideum*.

We model cells as self-propelled soft disks of radius 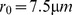. For each cell 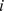 we specify the location of its center and its orientation by the two-dimensional vectors 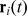 and 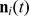 (by definition 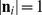). We specify locations of the cells using a rectangular 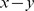 coordinate system, where the chamber in which the cells move is located in 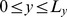. In the experiment, the chamber boundaries, 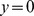 and 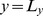, have perforations and are thus permeable to transport of cells and cAMP. The speed of each cell 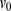 is assumed to be well-approximated as constant in time 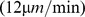, independent of signal strength, in agreement with controlled chemotaxis experiments [6]. The cAMP concentration field is denoted 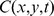. In the experiment the cells are deposited in a large reservoir (corresponding to 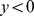 in the model) where there is no externally injected cAMP. This experimental condition is modeled by a Dirichlet boundary condition on the cAMP concentration, 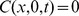 at 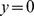, and by introducing individual discrete cells at 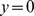 with a uniform flux 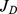 cells per unit time per unit length in 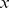 (each newly introduced cell's orientation is initially in the 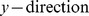). In addition, the experiment has an aqueous solution of cAMP in a large reservoir on the other side of the chamber (corresponding to 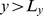 in the model), and the cAMP concentration in this reservoir stays constant during the course of the experiment. This is modeled by a Dirichlet boundary condition at 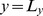, 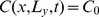, along with the removal of cells when they reach 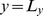. We applied periodic boundary conditions in 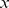, such that 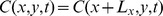 and each cell that leaves the chamber at a lateral boundary, 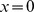 or at 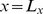, reenters the chamber at the other end. Using these definitions, we propose the following minimal, agent-based model for cell motion in our experimental setup,
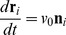
(1)

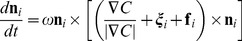
(2)

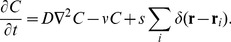
(3)


The first equation corresponds to the constant speed assumption.

The second equation dictates that the unit vector specifying the cell's orientation 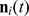 is attracted toward the direction of the vector,
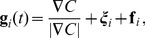
(4)with relaxation time 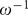. This relaxation time may be thought of as including both the chemically determined time for a cell to ‘perceive’ the gradient, as well as the time it takes the cell to mechanically turn its orientation. The first term in 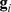 is a unit vector in the direction of the cAMP gradient. Note that, in accord with the observed similarity of the second, third, and fourth panels of Fig. 1D, this term is independent of the level of cAMP (i.e., invariant to the transformation 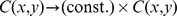). The second term 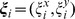 in 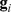 is white noise,
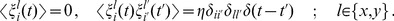
(5)The third term 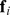 in 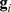 is a repulsive ‘force’ modeling a soft two-body contact interaction between neighboring cells,
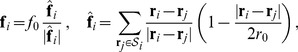
(6)where 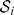 is the region 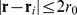. In Eq. (6) we have taken the form of the repulsive force to decrease linearly with distance from the center of the cell. We have also tried other forms for the 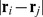 dependence of the repulsive force and found that no qualitative differences occurred. Szabo et al. [57] and Chate et al., [58] discuss the effect of adding cohesive (i.e., attractive) forces in modeling tissue cells. The parameter 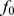 determines the strength of the repulsion force.

Eq.(3) is the diffusion equation governing the evolution of the distribution of the cAMP density, with constant diffusivity 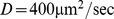 [59]. The parameter 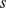 is the cAMP secretion rate of a cell. The cAMP decays at a rate 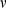 which can be spatially nonuniform and is approximately proportional to the concentration of the degradation enzyme phosphodiesterase PDE1 [25]. We introduce a degradation inhomogeneity suitable for our experimental setup in the following section.

### cAMP degradation has a non-linear profile due to the experimental conditions

The cAMP degradation rate 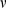 in Eq. (3) is meant to account for the presence of the cAMP degrading enzyme PDE1 with 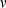 assumed proportional to the enzyme density 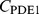. Since PDE1 is secreted by the cells themselves and then diffuses, we can expect that 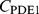 and hence 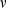 are time and space dependent quantities obeying an equation similar to Eq. (3) for the cAMP density 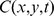, but with the term analogous to the degradation in Eq. (3) omitted. In the interest of simplicity, for our minimalist model, we wish to circumvent a full time-dependent diffusion equation model for 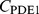. Instead, we assume that a time-independent steady state that is homogeneous in 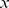 is established for the 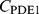 (we show in Text S1 that this is justified for the conditions of our experimental setup). This corresponds to 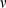 depending on 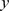 but not 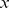 and 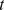, 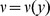. Furthermore, in steady state, the 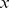-averaged cell flux in the 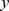-direction must, by conservation of cell number, be independent of 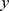 in the linear gradient chamber, and its value everywhere in the chamber must be the same as the cell injection flux 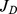 at 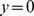. In the simplest case, without clumps, the 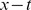 averaged density of cells in the external linear gradient region will thus be roughly uniform in 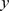 and of the order of 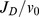. Thus the 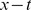 averaged PDE1 density 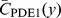, satisfies a one-dimensional, time-independent diffusion equation of the form
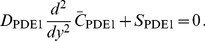
(7)Here we approximate 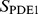 as constant in 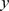 and given by 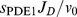 where 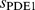 is the production rate of the PDE1 per cell per unit time; 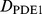 is the diffusivity of the PDE1 and is approximately 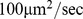 [60]. In addition, we will argue that the appropriate boundary conditions on the PDE1 density are 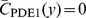 at 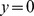 and 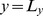. Solution of Eq.(7) with these boundary conditions leads to the model,
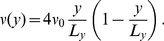
(8)That is, 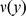 varies parabolically in 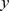; 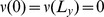, and has its maximum value 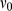 in the center of the chamber, 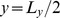. In our numerical explorations we mostly use the model Eq. (8). We also note that in other experiments, depending on the experimental setup, 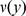 may have different dependence on 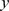. For comparison, we repeated our numerical runs with the spatially constant form 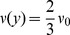, where the numerical prefactor 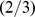 is chosen so that the total amount of PDE1 in 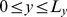 is the same as for Eq. (8) (i.e., 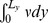 is the same). The spatially constant form for 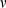 was used in other models of *D. Discoideum* chemotaxis [9], [21], [22], [24]. The results (shown in Text S1) are qualitatively similar to the results presented here.

We now outline how we motivate the use of the boundary conditions 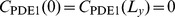 (more detailed quantitative justification is given in Text S1). In our experiments, cells are placed in the reservoir located in 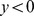. The cells then rapidly sink to the bottom of the reservoir (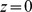). The reservoir has a vertical thickness that is more than 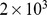 times larger than the vertical thickness of the linear gradient chamber. The same dimensions apply for the reservoir in 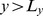. The bottom glass surface (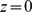) of the reservoir in 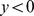 extends into 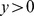, where it forms the bottom plane of the linear gradient chamber and of the reservoir in 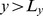. Cells that are on the bottom of the 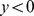 reservoir supply a source of cells for entry at 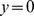 into the linear gradient chamber. The cAMP-degrading-enzyme PDE1, secreted by cells in the 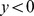 reservoir are assumed to be transported vertically upward by small convection flows in the reservoir fluid into the vertically large region 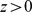 of the reservoir. In contrast, the distribution of the PDE1 emitted by the cells in 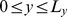 is constrained to the much thinner vertical region defined by the chamber dimensions. Thus, in the linear gradient chamber the PDE1 density cannot be attenuated to low levels by spreading vertically. As quantitatively shown in Text S1, based on this consideration, the enzyme density in 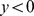 and 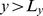 is much less than in the interior of the chamber. This leads to our previously stated approximate boundary conditions, 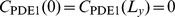, used in obtaining Eq. (8).

### Normalization of parameters

In order to systematically determine the essential dependence of the behavior of the model on its parameters, we introduce appropriate nondimensionalizations. We define the dimensionless spatial coordinates 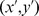 by 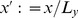 and 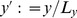. The dimensionless time scale 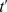 is defined as 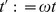, and the dimensionless cAMP density 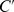 is defined as 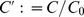. With the rescaled variables, the cAMP boundary conditions become, 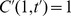 and 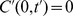. Additionally, the white noise is transformed to 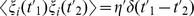, where 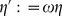. The model equations with the rescaled variables and Eq. (8) for 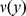 can now be written as
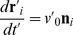
(9)

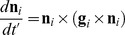
(10)

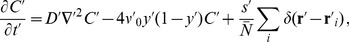
(11)where 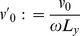, 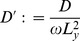, 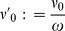, 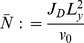, and 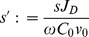. The integral of the summation 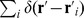 over the square 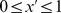, 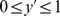 is the number of cells in the unnormalized square 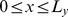, 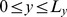 and is roughly 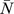. In the situations we investigate 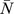 is always large compared to unity. Thus the term 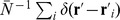 roughly plays the role of a normalized density whose nominal value is one. With these normalizations, the parameters in our model are 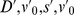, 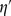, and 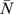. We wish to explore the variation of the system behavior as a function of parameters. This is clearly an impossible task to carry out for the full set of 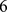 dimensionless parameters just listed. Thus we now seek to restrict our detailed considerations to just a few of these parameters whose influence is, we think, the most interesting. If we regard 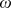 for the cells as fixed, then the parameter 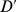 is dictated by the experimental setup. Experimentally, the typical cell speed 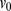 and hence 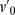 is observed to be roughly the same for wild type, and mutant cells [6], and we therefore take 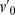 as fixed. The noise term 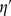 will be fixed by the experimental observations (e.g., Fig. 1D) which imply that it does not vary significantly across the different experimental conditions investigated (see Text S1). Thus we will keep 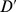, 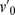 and 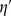 fixed at the appropriate estimated values. Furthermore, we suspect that the qualitative behavior of the system will be insensitive to the precise value of 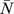 so long as 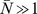 (the situation we are interested in). Thus our main numerical model explorations will focus on how the model behavior depends on 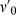 and 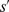.

We now further discuss our reason for interest in varying 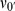 and 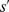. First, with respect to 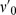, in reference [8] a genetic perturbation to the cells results in mutants lacking the ability to produce the degradation enzyme PDE1 (but still emitting cAMP). In our model this corresponds to setting 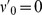. In our numerical experiments we will explore a continuous dependence on 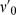, partly because 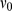 is not well determined, but also to understand the difference between mutant cells that do not emit PDE1 (i.e., pdsA-/PEC cells) and wild-type cells. We also suggest that it may be useful for future experiments to explore continuous dependence on PDE1 secretion rate (i.e., 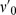) which might be realized by introducing a mixture of wild-type and mutant PDE1- cells. Regarding variations of 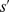, we note that the secretion of cAMP from cells 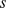, is biologically inhibited for another type of mutant, the aca- cells. Also, in our experiments, we change the external concentration of cAMP, 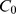. The biological and chemical changing of the parameters, 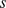 and 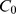, both yield change of 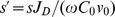. (Also, 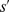 could be tuned by changing the 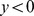 reservoir cell density and hence 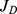, but we have kept 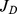 constant in our experiments.)

### Parameters

Aside from 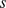 and 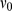 the parameters we used in our simulations are summarized in Table 1. We assume that the cell parameters in this table (i.e., 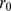, 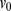, 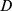, 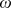, 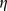, 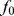) are the same for wild-type cells (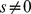) and mutant cells (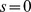). In the absence of mutual attractions through cell's secretion of cAMP, a Fokker-Planck version of Eqs.(1–6) can be solved analytically (see the Text S1), and 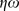 in Eq. (5) can be determined by matching the analytical result to experimental observations of mutant cells. Also, we estimate 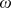 as being of the order of 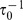 determined from our experimentally observed time-autocorrelation of the orientation vector (Fig. 2A), where 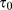 is defined at the end of the previous section. This time scale is comparable to the contraction rate of *D. discoideum* cells which in the work of Satulovsky et al. [31] is considered as the bulk relaxation time. We note that the real cells' secretion rates of cAMP and of PDE1 are not well quantified and can be varied by drug treatment or by the use of mutant cells. Thus we will regard 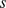 and the PDE1-level-dependent parameter 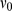 as variable parameters and investigate how the dependence of the collective cell dynamics depends on them.

**Table 1:** Simulation parameters.

Symbol	Description	Value
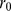	Cell radius	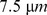
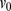	Self-propulsion speed	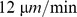
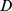	Diffusion constant of cAMP	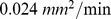
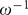	Response time	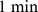
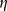	Amplitude of Gaussian white noise	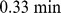
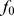	Repulsive force constant (dimensionless)	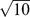
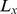	Width of the simulation box	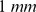
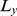	Length of the simulation box	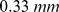

Parameters used in the numerical simulations. Except for the force constant 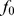, all the cell parameters in this table (i.e., 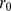, 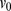, 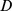, 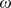 and 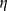) are obtained from experiments. The response time is obtained from the autocorrelations of the displacement vector. The noise amplitude 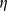 was calculated from the variance of the 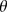 distribution, where the angle 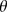 represents the orientation of the associated displacement vector.

### Results of numerical simulations capture experimentally observed migration patterns

**Figure 3:**
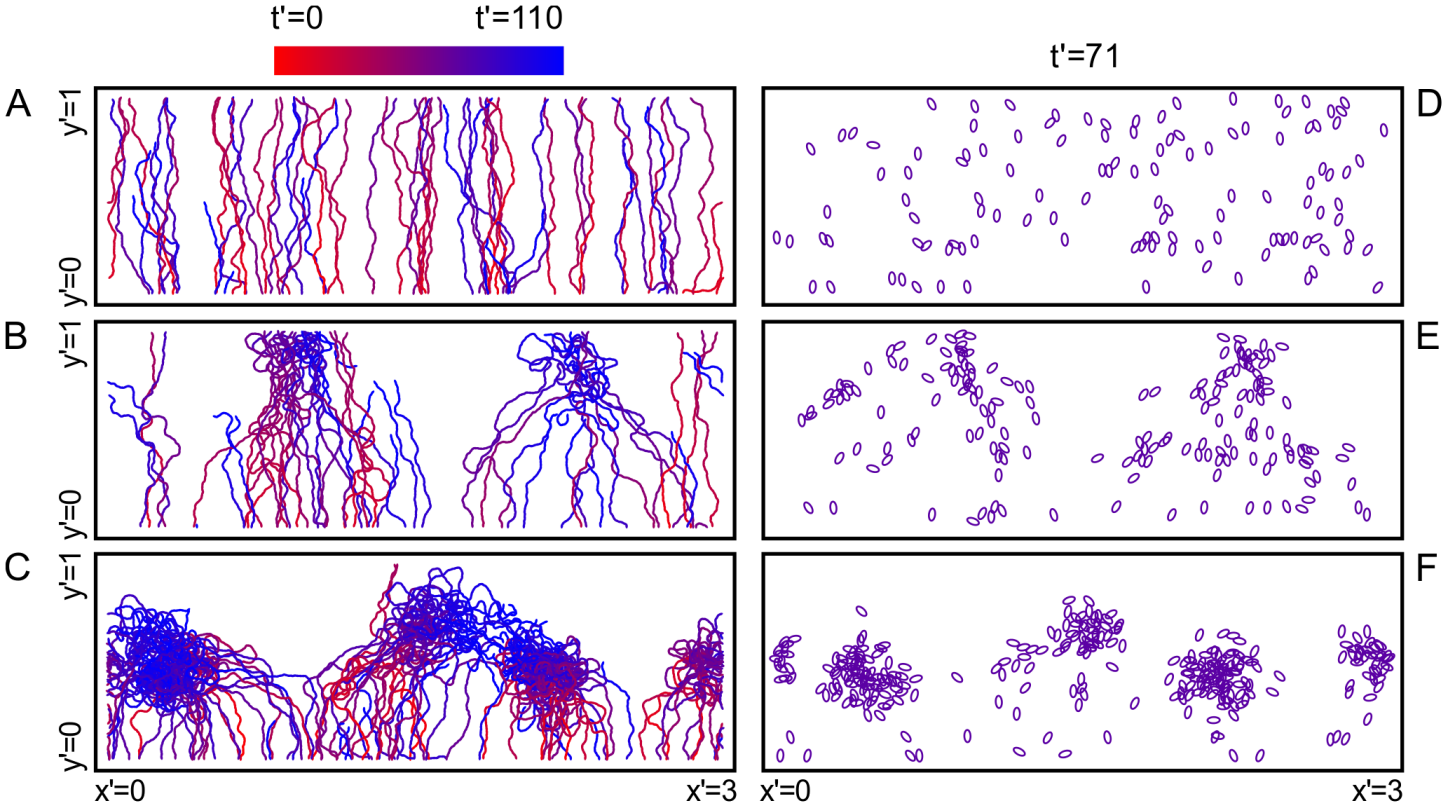
Cell tracks from simulations for the three representative modes of collective motion, uncorrelated motion, streaming, and aggregation. (A) For a relatively slow cAMP secretion rate (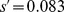) the cells move independently, showing no sign of collective motion. (B) If the cAMP secretion is moderate (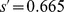) cells form streams. (C) For high relative cAMP secretion rate (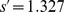) cells exhibit aggregation and therefore form clumps. Figs. (D–F) are snapshots from the same simulations exhibiting the spatial organization of the cells.

The model equations, Eqs.(1–6) are simulated numerically. Figs. 3A–3C show representative cell tracks for three different values of the normalized cAMP secretion rate 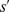. For all three of these cases 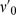 is fixed at 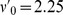, which we estimate to be consistent with previous experimental measurements [21]. The color at a given point on a cell track in Figs. 3A–3C indicates the time that the cell making the track was at that point, where red corresponds to the beginning of the simulation and blue corresponds to the end of the simulation. Figs. 3D–3F show representative snapshots, where the position and the orientation 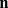 of the cell is indicated by an ellipse (at normalized time 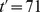 for D, E, and F). In the top panels of Fig. 3 (Figs. 3A and 3D), the relative cAMP secretion rate is very small (i.e., 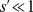). This regime mimics the aca- mutant cells, and our numerical results qualitatively agree with the experimental observations of aca- cells (cf., 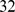 min panel of Fig. 1B). For larger values of 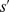, and depending on 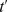, our numerical results can be classified under two main categories, streams (Fig. 3E) and clumps (Fig. 3F). At moderate 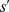 (Fig. 3E) streams are evident. At higher 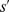, Fig. 3F shows that multiple clumps of cells form. From the corresponding tracks of cells shown in Fig. 3C, it is seen that the cells stay within the clumps and the clumps have almost no motion in the 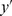 direction.

### Dynamics of collective migration is quantified by the mean progression speed and cell density

To go beyond the visual comparison of our simulation results with our experimental observations, a quantitative description of the three modes of group cell motion described above (i.e., uncorrelated motion, streams, and clumps) is desirable. We define the normalized mean progression 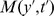, by 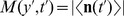, where the angle brackets denote an average of cells in the region between 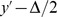 and 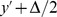, where 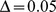 (cf., [61], [62]). We denote by 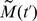 the average of 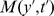 over 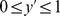, and we denote by 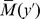 the time average of 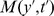 taken over the last quarter of the simulation (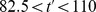). Another useful measure is the normalized averaged cell density 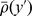, computed by averaging over the region 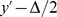 to 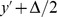 with 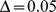 and normalized so that 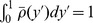.

**Figure 4:**
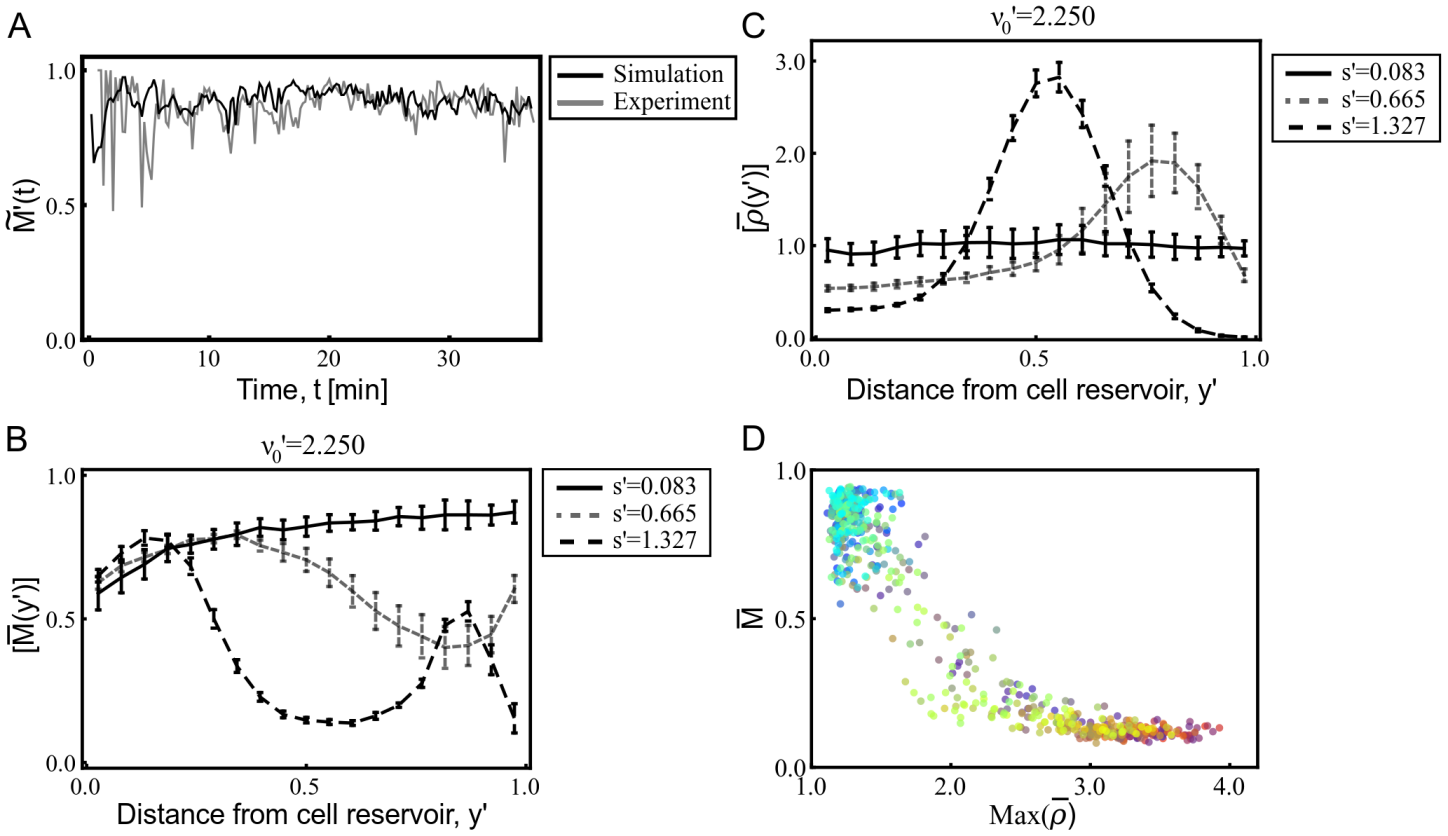
Mean progression speed and the cell density are used in quantifying collective motion. (A) 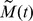 is used to compare experimental data (aca- with 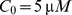) with a representative single run that is obtained with model parameters that mimic the experimented aca- mutant cells. (B) and (C) show respectively, 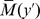, and 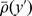 as a function of the distance from the cell reservoir for 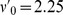, and three different cAMP secretion rates. Error bars are obtained from different realizations with the same simulation parameters for each curve and represent the standard error of the mean. (D) The maximum 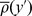 in the region 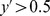 is plotted against its corresponding 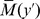. Each point corresponds to a single numerical run. For (A), when the cells enter the chamber at 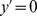, we initialize the cell orientation vectors 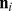 for cell 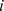 according to a distribution of the angle 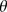 with respect to the 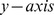, where this distribution is uniform in the range, 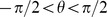. This is done so as to roughly match the experimental 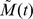 at 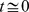.

First, Fig. 4A shows the ensemble average of 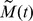, denoted by 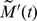, for the aca- cell experiment (in gray) and for a single model simulation (in black). The model parameters for the run are 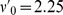 and 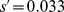, which correspond to the aca- mutant cells. To make a fair comparison, for the experimentally obtained 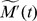 we filtered out cells that move at a slower speed than what we considered in our model (i.e., 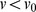). We calculate 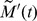 for a group of randomly selected cells in the 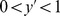 region. Since our tracking algorithm cannot track all the cells available in this region, the experimentally obtained 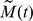 is represented by this ensemble average. To compare our experimental result to our numerical simulation results, we calculate 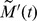 from our simulation by sampling cells in the simulation so as to match the number of cells for which 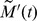 is experimentally calculated.

We show in Figs. 4B and 4C how 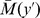, and 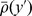 vary with the distance from the cell reservoir, 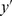, for the three values of 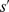 used to obtain the cell tracks shown in Fig. 3 with 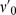 fixed at the same value used for Fig. 3. In these plots, 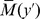, and 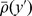 are averaged over several runs (this average is denoted by 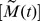), where the error in the mean is shown by vertical error bars, which is calculated by the standard deviations of the runs divided by the square root of the number of runs. In the low 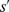 regime (solid curves in Figs. 4B and 4C), corresponding to Figs. 3A and 3D, Fig. 4A shows that, 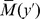 saturates to 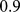 in the upper half of the gradient chamber, 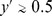, while Fig. 4B shows that 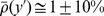 is approximately uniform. The density profiles measured from the time lapse images (a rough estimate calculated from the image intensity) fairly agree with those obtained from our simulations. For PDE1- cells, our model suggests that the cAMP secretion levels are small compared to the wild-type cells exposed to the same imposed gradient. The density profiles measured from the time lapse images (a rough estimate calculated from the image intensity) fairly agree with those obtained from our simulations. For PDE1- mutant cells, our model suggests that the cAMP secretion levels are small compared to the wild-type cells exposed to the same imposed gradient. In determining the cAMP secretion rate we assumed same noise level compared to the wild-type cells. Therefore, in conjunction with findings from our model, our experimental observations suggest that the lack of degradation of external cAMP results in either reduced signal relay or increased noise level in gradient sensing (corresponding to receptor desensitization). The comparison and the details of the density estimate are shown in Text S1.

As shown in Figs. 3B and 3E, for 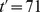, streams emerge in the regime of moderate 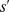 (plotted as the gray dashed curves in Figs. 4B and 4C). These streams start to aggregate in the upper half of the gradient chamber, and this results in a decrease in 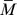 and a corresponding increase in 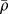. Compared to the low 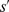 regime, the streams cause an increase in the cell density (the peak at 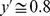).

In the high 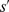 regime (plotted as the black dashed curves in Figs. 4B and 4C), 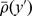 is even more peaked than in the moderate 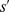 regime. This apparently leads to a peak in the cAMP density which leads cells to start aggregating in the lower half of the gradient chamber. Streams form close to the reservoir, where cells enter the gradient chamber. To form streams, newly entering cells acquire laterally (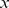-directed) converging velocity components. Since the cell speeds are fixed at 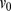, this causes 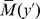 to decrease (see the region 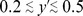 in Fig. 4B) and 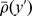 to increase. This apparently leads to a more localized secretion of cAMP, which overcomes the externally imposed cAMP concentration causing the clumping seen in Figs. 3C and 3F.

In Fig. 4D the maximum 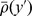 in the region 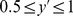 is plotted versus the corresponding 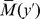. Each point in this figure is obtained from a single numerical run. The points are color coded with respect to the 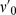 and 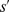 used in the numerical run. Fig. 4D shows that points are clustered in two regions. The first region, where 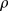 is large and 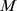 is small [(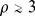), 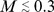], corresponds to large clumps, while the second region, where 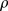 is small and 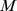 is large [(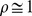), 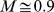], corresponds to the uncorrelated motion. The points between these two regions correspond to runs where cells form streams which either generate clumps (i.e., points closer to the first region) or move through the 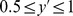 region and leave the gradient chamber (i.e., points closer to the second region).

### Stream formation is robust when external cAMP is degraded

**Figure 5:**
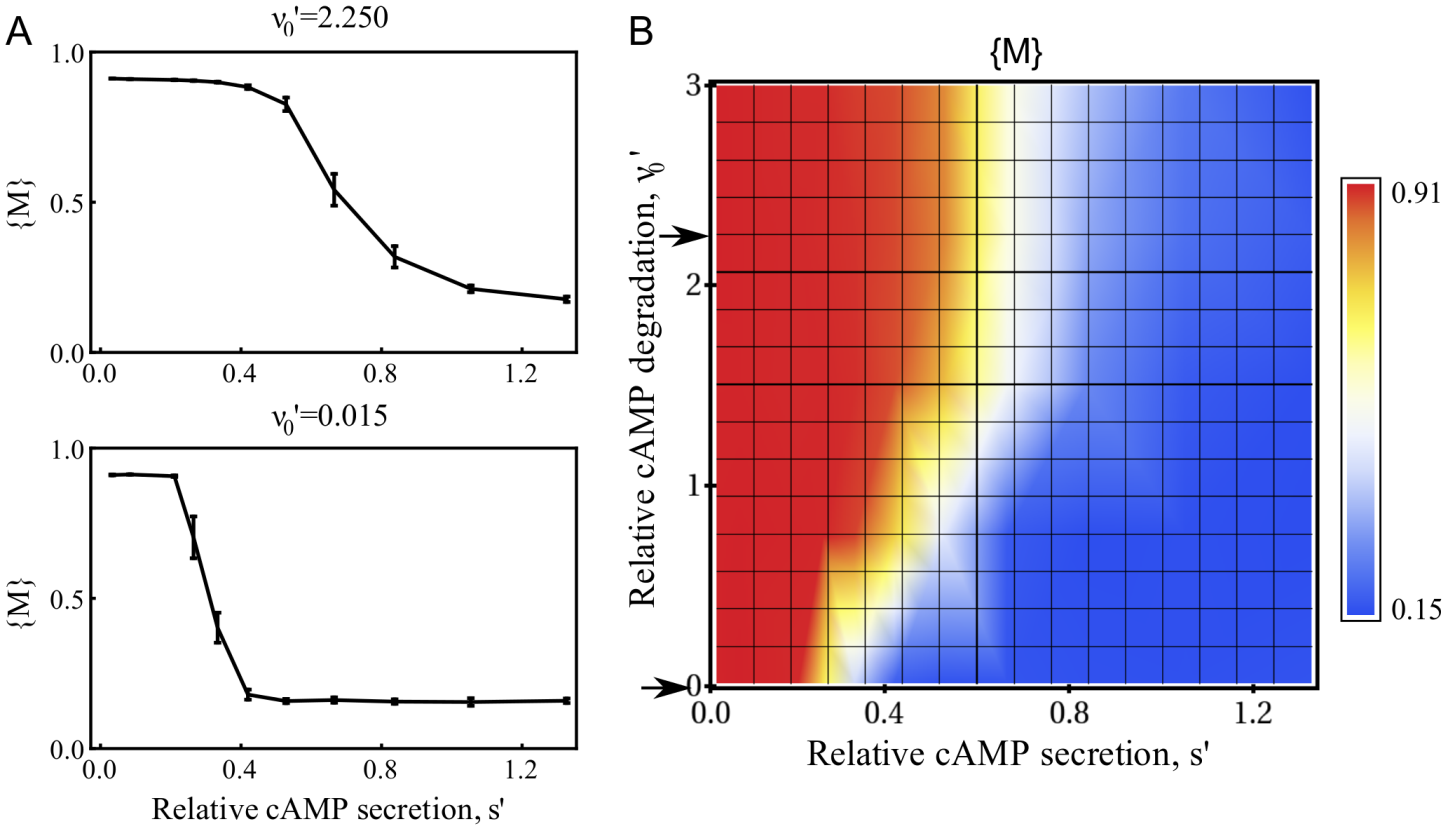
Mean progression 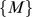, as a function of relative signaling rate 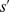, and relative degradation rate 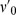. (A) 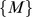 as a function of 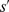. Error bars are obtained from many numerical realizations (between 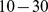) and represent the standard error of the mean. In the top panel, the degradation rate is comparable to the experimentally obtained degradation of the phosphodiesterase. In the bottom panel, we used small cAMP degradation rate, which models the mutant PDE1- cells, incapable of secreting the enzyme that degrades cAMP. (B) 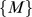 as a function of the relative cAMP secretion and relative cAMP degradation rates. The red regions correspond to uncorrelated motion. The dynamically unstable regions, where streams are likely to form, of the (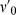, 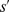) phase space is labeled with yellow and white. Blue regions are associated with aggregate formation.

In our model there are two time scales, 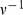 and 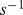 (the cAMP degradation rate and the local cAMP production rate), and we explored their effects. Fig. 5 shows results for 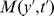 averaged over 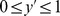 and 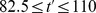 (i.e., the last quarter of the simulation), as well as over a large number of model simulations (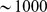). These averages are labeled 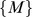 in the figure. The top panel of Fig. 5A shows 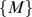 as a function of 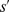 for 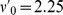. Fig. 5A shows that 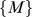 decreases as 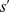 increases. In the region 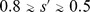, where 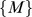 decreases fastest, streams occur, but clumps are rare (e.g., Figs. 3B and 3E). The bottom panel of Fig. 5A is for a very small value of 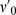 (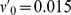), modeling mutant cells that cannot degrade cAMP. In this case we see that there is a sharp decrease in 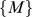 in the range 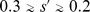. Below this range the simulations show roughly uniform cell density, while above this range clumps occur. Compared to the slow degradation regime, in the fast degradation regime (top panel of Fig. 5A) the streaming behavior is robust. In the slow degradation regime, the streams form for only a short period which is followed by formation of clumps. Recent experiments demonstrate that stream formation is impaired, if cells cannot degrade external cAMP [8]. Fig. 5B summarizes results for our simulations (color coded), as a function of 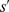 (plotted on the horizontal axis) and 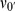 (plotted on the vertical axis). The data in the top (bottom) panel of Fig. 5A corresponds to a horizontal cut through Fig. 5B at the arrow, 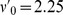 (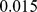), on the vertical axis of Fig. 5B. Fig. 5B shows that the width of the range of 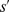, where streams occur, decreases as 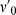 is lowered. Additionally, the onset of stream generation with respect to 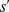 becomes smaller with decreasing 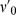.

## Discussion

Our model explains different observed modes of collective motion of motile cells. Our main new finding is that signal relay alone is not enough to arrange migrating cells into collectively moving streams. However, when the signal is not only relayed but also degraded, stable streams form. Our model is minimal, involving a relatively small number of potentially experimentally deducible parameters.

Based on our numerical results, we suggest experiments where the transition between streaming and clumping can be experimentally tested by changing the effective values of our model parameters. One suggestion is that the value of 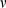 can be effectively reduced by either mixing wild-type and PDE1- mutants or by changing the amount of PDE1 added during the PDE1- mutant cell development.

The relaxation time 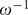, obtained from our experimental observations, is associated with the membrane retraction time scale. In addition, the time scale corresponding to the noise amplitude 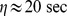 is associated with the formation time of pseudopods [63]. These parameters could be altered by adding drugs or changing the developmental procedures. For example, introducing a drug that inhibits the PI3 kinase severely reduces the pseudopod generation frequency [63] and hence both 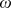 and 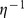. Additionally, recent studies show drastic change in the collective motion behavior of wild type cells when they are prepared over a longer development time [64]. In this case 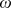 and 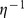 are reduced in agreement with the observed reduction of stream formation [64]. Thus, we believe that our model can be utilized to quantify changes in the collective motion in response to modifications of cell characteristics.

In our model, we have only focused on the extracellular cAMP dynamics given in Eq. (3) with the objective of reproducing the patterns in Fig. 1 with as few physical processes as possible. We modeled the motion of the cells according to the the dynamics of sensing the signal with the phenomenological equation Eq. (2). Models that include additional processes (not included in our model) are capable of explaining additional phenomena. E.g., models of cAMP signal transduction including receptor desensitization [21] and adaptation [55] show the generation of experimentally observed cAMP waves including spiral waves [3], [9], [56]. In addition, the observed rotating vortex structure of the aggregates can be explained by other self-propelled particle models which allow cells to adjust their propulsive force [65]. In the future we plan to modifying our model to investigate the effect of dynamic cell-cell adhesion in stabilizing stream formation, and aggregation.

Our model can be extended to include competition between the gradient steepness, 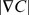, and the local cAMP concentration, 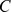, by modifying Eq. (4) and introducing a competition between the noise intensity and the concentration of the cAMP. A simple approach is to impose the following limits: For small local cAMP concentration, the noise (second term in Eq. (4)) will have a higher effect in the directionality (i.e. independent random motion). In contrast, for high local cAMP concentration, the contribution from the noise to local cAMP concentration ratio should be small compared to the gradient steepness to local cAMP concentration ratio. When the model is extended to include this competition, we can define an organization time scale as a measure of cellular organization. Thus, we can measure the efficiency of stream formation not only with respect to signal relay but also with respect to the efficiency of directional sensing.

We believe that our simplified approach, used here for *D. discoideum* can be extended to more complex cells exhibiting signal relay, such as neutrophils [49], [66]. For neutrophils, signal relay is less well understood [49]. However, our numerical simulations can be utilized to distinguish uncorrelated motion from weak signal relay: Using our simulations in conjunction with linear gradient experiments where cells do not converge naturally to an external signal, we can calculate the effect of signal relay on the mean progression speed, as well as the development of an inhomogeneous density due to cell-cell attraction, even in the case of very small signal relay that is not sufficient to lead to discernible clumps or streams. Moreover, our model can be potentially extended to include the dependence of signal relay on cell density, in order to compare the dynamics to those observed in Ref. [67], which proposes a quorum sensing mechanism that can quantify the persistent random walk of *D. discoideum* at different phases of development as well as different densities. Another potential use of our model is to model migration when subpopulations of cells have different signal sensing, and signal relay capabilities. A prominent example of such collective migration is the motion of neural crest cells, a collective process during embryonic development. Recent experiments suggest that mathematical models of the neural crest migration require subpopulations having different chemotactic responses [68].

## Methods

### Experiments in linear cAMP gradient

To examine the chemotactic dose response, cell migration was recorded at 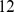 second intervals for 1 hour in the EZ-TAXIScan chamber (Effector Cell Institute, Tokyo, Japan). In the absence of wild-type cells the device establishes a well-defined, stable cAMP gradient during the course of the experiment [47]. *Dictyostelium discoideum* cells, wild-type cells (ax3) and its ACA null mutant cells (aca-) were prepared as described previously in Ref. [6]. PDE1- cells were prepared as described previously in Ref. [8].

### Computational implementation

There are two modules in our numerical simulation code, the first module consists of the equations of motion given in Eqs. (1)–(3) which defines the position and the direction of motion of cells based on the local gradient in the neighborhood of each cell. The second module calculates the diffusive time evolution of cAMP due to the external signal and dynamic local intercellular signals and provides the updated gradient vector field for use in the first module. Simultaneous evaluation of these two modules generates cell tracks. The diffusion equation (Eq. (3)) for the cAMP is solved explicitly on a square grid with spacing 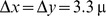
m using a forward time and central space Euler method. In the numerical simulations the time step is 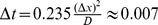 seconds, which is well in the stable range of the numerical algorithm. For implementing the numerical evaluation of 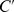 the diffusion equation is discretized with 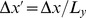 and 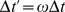. The Laplace operator can be replaced by the discretized Laplace operator and the Dirac-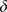 function is discretized in one dimension as 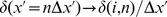, where 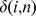 is the Kronecker-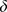 function, it is zero except for 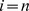. Thus, the value of the cAMP field at 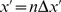 and 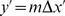, where 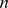 and 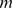 are integers, is updated according to
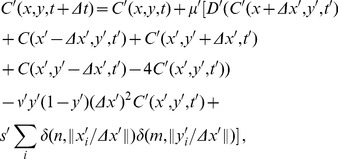
(12)with 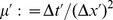. In Eq. (12), 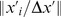 rounds its argument to the nearest integer. The same 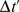 is used in evaluating the equations of motion (Eqs. (1) and (2)). Table 1 shows the definitions and values of the parameters used in the numerical simulations.

## Supporting Information

Figure S1
**Results for the uniform cAMP degradation scheme.** (A) The degradation rate as a function of the distance from the cell reservoir, where 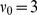. (B) 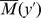 is shown for three representative relative cAMP secretion rates, whose dynamics is shown in Fig.3. (C) 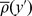 for the same relative cAMP secretion rates used in the upper panel. (D) Maximum 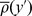 in the 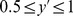 region, is plotted against its corresponding 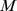 for all numerical simulations with constant degradation scheme. Each point represents a single numerical realization and is color coded with respect to 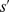. (E) 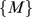 is plotted against 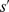, where the each data point is obtained from averaging many numerical realizations 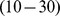. The vertical bars represent the error in the mean, which is calculated by the standard error from many realizations.(TIF)

Figure S2
**Density profile measurements.** The density, 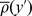, is plotted against the distance from the cell reservoir for wild-type cells moving in low cAMP concentration in the reservoir (left), wild-type cells moving in high cAMP concentration in the reservoir (center) and aca- mutant cells moving in high cAMP concentration in the reservoir (right). The density profile is obtained both from experiments and simulations of the model for (A) 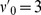, 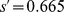, (B) 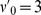, 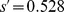, (C) 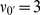, 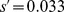, (D) 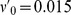, 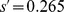. Each simulation data point is obtained from averaging many numerical realizations. The vertical bars in both experimental and simulation data represent the standard error of the mean.(TIF)

Text S1 **The supplementary text provides details regarding the assumptions used in our model in addition to comparison of numerical results with experimental observations.**
(PDF)

## References

[1] Parent CA, Devreotes PN (1999) A cell's sense of direction. Science 284: 765–70. 10221901 10.1126/science.284.5415.765

[2] Schienbein M, Gruler H (1993) Langevin equation, Fokker-Planck equation and cell migration. B Math Biol 55: 585–608. 10.1007/BF02460652 8364419

[3] van Oss C, Panfilov AV, Hogeweg P, Siegert F, Weijer CJ (1996) Spatial pattern formation during aggregation of the slime mould Dictyostelium discoideum. J Theor Biol 181: 203–13. 8869123 10.1006/jtbi.1996.0126

[4] Hu B, Fuller D, Loomis WF, Levine H, Rappel WJ (2010) Phenomenological approach to eukaryotic chemotactic efficiency. Phys Rev E Stat Nonlin Soft Matter Phys 81: 031906. 20365769 10.1103/PhysRevE.81.031906 PMC2872938

[5] Gregor T, Fujimoto K, Masaki N, Sawai S (2010) The onset of collective behavior in social amoebae. Science 328: 1021–5. 20413456 10.1126/science.1183415 PMC3120019

[6] McCann CP, Kriebel PW, Parent CA, Losert W (2010) Cell speed, persistence and information transmission during signal relay and collective migration. J Cell Sci 123: 1724–31. 20427323 10.1242/jcs.060137 PMC2864714

[7] Driscoll MK, McCann C, Kopace R, Homan T, Fourkas JT, et al (2012) Cell shape dynamics: from waves to migration. PLoS Comput Biol 8: e1002392. 22438794 10.1371/journal.pcbi.1002392 PMC3305346

[8] Garcia G, Rericha E, Heger C, Goldsmith P, Parent C (2009) The group migration of dictyostelium cells is regulated by extracellular chemoattractant degradation. Mol Biol Cell 20: 3295–3304. 19477920 10.1091/mbc.E09-03-0223 PMC2710833

[9] Levine H, Aranson I, Tsimring L, Truong TV (1996) Positive genetic feedback governs cAMP spiral wave formation in Dictyostelium. Proc Natl Acad Sci U S A 93: 6382–6. 8692824 10.1073/pnas.93.13.6382 PMC39031

[10] Sawai S, Thomason PA, Cox EC (2005) An autoregulatory circuit for long-range self-organization in Dictyostelium cell populations. Nature 433: 323–6. 15662425 10.1038/nature03228

[11] Chang YY (1968) Cyclic 3′,5′-adenosine monophosphate phosphodiesterase produced by the slime mold Dictyostelium discoideum. Science 161: 57–9. 4298142 10.1126/science.161.3836.57

[12] Gerisch G, Hülser D, Malchow D, Wick U (1975) Cell communication by periodic cyclic-AMP pulses. Philos Trans R Soc Lond, B, Biol Sci 272: 181–92. 1814 10.1098/rstb.1975.0080

[13] Devreotes PN, Derstine PL, Steck TL (1979) Cyclic 3′,5′ AMP relay in Dictyostelium discoideum. I. A technique to monitor responses to controlled stimuli. J Cell Biol 80: 291–9. 222769 10.1083/jcb.80.2.291 PMC2110346

[14] Kriebel P, Barr V, Parent C (2003) Adenylyl cyclase localization regulates streaming during chemotaxis. Cell 112: 549–560. 12600317 10.1016/s0092-8674(03)00081-3

[15] Potel MJ, Mackay SA (1979) Preaggregative cell motion in Dictyostelium. J Cell Sci 36: 281–309. 457811 10.1242/jcs.36.1.281

[16] Fürth R (1920) Die Brownsche Bewegung bei Berücksichtigung einer Persistenz der Bewegungsrichtung. Mit Anwendungen auf die Bewegung lebender Infusorien. Zeitschrift für Physik 2: 244–256.

[17] Gail MH, Boone CW (1970) The locomotion of mouse fibroblasts in tissue culture. Biophys J 10: 980–93. 5531614 10.1016/S0006-3495(70)86347-0 PMC1367974

[18] Futrelle RP, Traut J, McKee WG (1982) Cell behavior in Dictyostelium discoideum: preaggregation response to localized cyclic AMP pulses. J Cell Biol 92: 807–21. 6282894 10.1083/jcb.92.3.807 PMC2112044

[19] Gingle AR (1976) Critical density for relaying in Dictyostelium discoideum and its relation to phosphodiesterase secretion into the extracellular medium. J Cell Sci 20: 1–20. 175073 10.1242/jcs.20.1.1

[20] Gingle AR, Robertson A (1976) The development of the relaying competence in Dictyostelium discoideum. J Cell Sci 20: 21–7. 175075 10.1242/jcs.20.1.21

[21] Martiel JL, Goldbeter A (1987) A Model Based on Receptor Desensitization for Cyclic AMP Signaling in Dictyostelium Cells. Biophys J 52: 807–28. 19431710 10.1016/S0006-3495(87)83275-7 PMC1330185

[22] Levine H, Reynolds W (1991) Streaming instability of aggregating slime mold amoebae. Phys Rev Lett 66: 2400–2403. 10043475 10.1103/PhysRevLett.66.2400

[23] Steinbock O, Siegert F, Muller SC, Weijer CJ (1993) Three-dimensional waves of excitation during Dictyostelium morphogenesis. Proc Natl Acad Sci U S A 90: 7332–5. 8394018 10.1073/pnas.90.15.7332 PMC47131

[24] Kessler DA, Levine (1993) Pattern formation in Dictyostelium via the dynamics of cooperative biological entities. Phys Rev E 48: 4801–4804. 10.1103/physreve.48.4801 9961163

[25] Palsson E, Cox EC (1996) Origin and evolution of circular waves and spirals in Dictyostelium discoideum territories. Proc Natl Acad Sci U S A 93: 1151–5. 8577731 10.1073/pnas.93.3.1151 PMC40047

[26] Postma M, Haastert PJV (2001) A diffusion-translocation model for gradient sensing by chemotactic cells. Biophys J 81: 1314–23. 11509347 10.1016/S0006-3495(01)75788-8 PMC1301612

[27] Levchenko A, Iglesias PA (2002) Models of eukaryotic gradient sensing: application to chemotaxis of amoebae and neutrophils. Biophys J 82: 50–63. 11751295 10.1016/S0006-3495(02)75373-3 PMC1302448

[28] Levine H, Kessler DA, Rappel WJ (2006) Directional sensing in eukaryotic chemotaxis: a balanced inactivation model. Proc Natl Acad Sci USA 103: 9761–6. 16782813 10.1073/pnas.0601302103 PMC1502527

[29] Palsson E, Othmer HG (2000) A model for individual and collective cell movement in Dictyostelium discoideum. Proc Natl Acad Sci U S A 97: 10448–53. 10984537 10.1073/pnas.97.19.10448 PMC27044

[30] Armstrong NJ, Painter KJ, Sherratt JA (2006) A continuum approach to modelling cell-cell adhesion. J Theor Biol 243: 98–113. 16860344 10.1016/j.jtbi.2006.05.030 PMC1941683

[31] Satulovsky J, Lui R, Wang Yl (2008) Exploring the control circuit of cell migration by mathematical modeling. Biophys J 94: 3671–83. 18199677 10.1529/biophysj.107.117002 PMC2292371

[32] Hecht I, Skoge ML, Charest PG, Ben-Jacob E, Firtel RA, et al (2011) Activated membrane patches guide chemotactic cell motility. PLoS Comput Biol 7: e1002044. 21738453 10.1371/journal.pcbi.1002044 PMC3127810

[33] Schienbein M, Franke K, Gruler H (1994) Random walk and directed movement: Comparison between inert particles and self-organized molecular machines. Phys Rev E Stat Phys Plasmas Fluids Relat Interdiscip Topics 49: 5462–5471. 9961873 10.1103/physreve.49.5462

[34] Shenderov AD, Sheetz MP (1997) Inversely correlated cycles in speed and turning in an ameba: an oscillatory model of cell locomotion. Biophys J 72: 2382–9. 9129842 10.1016/S0006-3495(97)78883-0 PMC1184434

[35] Li L, Norrelykke SF, Cox EC (2008) Persistent cell motion in the absence of external signals: a search strategy for eukaryotic cells. PLoS One 3: e2093. 18461173 10.1371/journal.pone.0002093 PMC2358978

[36] Amselem G, Matthias T, Albert B, Eberhard B, Carsten B (2012) A Stochastic Description of Dictyostelium Chemotaxis. PLoS ONE 7: e37213. 22662138 10.1371/journal.pone.0037213 PMC3360683

[37] Selmeczi D, Mosler S, Hagedorn PH, Larsen NB, Flyvbjerg H (2005) Cell Motility as Persistent Random Motion: Theories from Experiments. Biophysical Journal 89: 912–931. 15951372 10.1529/biophysj.105.061150 PMC1366641

[38] Takagi H, Sato MJ, Yanagida T, Ueda M (2008) Functional analysis of spontaneous cell movement under different physiological conditions. PLoS ONE 3: e2648. 18612377 10.1371/journal.pone.0002648 PMC2444018

[39] Campos D, Méndez V, Llopis I (2010) Persistent random motion: uncovering cell migration dynamics. J Theor Biol 267: 526–34. 20858504 10.1016/j.jtbi.2010.09.022

[40] Bosgraaf L, Van Haastert PJM (2009) Navigation of chemotactic cells by parallel signaling to pseudopod persistence and orientation. PLoS One 4: e6842. 19718261 10.1371/journal.pone.0006842 PMC2729408

[41] Bosgraaf L, Van Haastert PJM (2009) The ordered extension of pseudopodia by amoeboid cells in the absence of external cues. PLoS One 4: e5253. 19384419 10.1371/journal.pone.0005253 PMC2668753

[42] Andrews BW, Iglesias PA (2007) An information-theoretic characterization of the optimal gradient sensing response of cells. PLoS Comput Biol 3: e153. 17676949 10.1371/journal.pcbi.0030153 PMC1937015

[43] van Haastert PJM, Postma M (2007) Biased random walk by stochastic fluctuations of chemoattractant-receptor interactions at the lower limit of detection. Biophys J 93: 1787–96. 17513372 10.1529/biophysj.107.104356 PMC1948060

[44] Ueda M, Shibata T (2007) Stochastic signal processing and transduction in chemotactic response of eukaryotic cells. Biophys J 93: 11–20. 17416630 10.1529/biophysj.106.100263 PMC1914446

[45] Fuller D, Chen W, Adler M, Groisman A, Levine H, et al (2010) External and internal constraints on eukaryotic chemotaxis. Proc Natl Acad Sci U S A 107: 9656–9. 20457897 10.1073/pnas.0911178107 PMC2906906

[46] Amselem G, Theves M, Bae A, Beta C, Bodenschatz E (2012) Control parameter description of eukaryotic chemotaxis. Phys Rev Lett 109: 108103. 23005333 10.1103/PhysRevLett.109.108103

[47] Kanegasaki S, Nomura Y, Nitta N, Akiyama S, Tamatani T, et al (2003) A novel optical assay system for the quantitative measurement of chemotaxis. J Immunol Methods 282: 1–11. 14604536 10.1016/j.jim.2003.07.008

[48] Bagorda A, Das S, Rericha EC, Chen D, Davidson J, et al (2009) Real-time measurements of cAMP production in live Dictyostelium cells. J Cell Sci 122: 3907–14. 19808889 10.1242/jcs.051987 PMC2773191

[49] Liu L, Das S, Losert W, Parent CA (2010) mTORC2 Regulates Neutrophil Chemotaxis in a cAMPand RhoA-Dependent Fashion. Dev Cell 19: 845–857. 21145500 10.1016/j.devcel.2010.11.004 PMC3071587

[50] Vinolo MAR, Ferguson GJ, Kulkarni S, Damoulakis G, Anderson K, et al (2011) SCFAs induce mouse neutrophil chemotaxis through the GPR43 receptor. PLoS ONE 6: e21205. 21698257 10.1371/journal.pone.0021205 PMC3115979

[51] Nitta N, Tsuchiya T, Yamauchi A, Tamatani T, Kanegasaki S (2007) Quantitative analysis of eosinophil chemotaxis tracked using a novel optical device TAXIScan. Journal of Immunological Methods 320: 155–163. 17289072 10.1016/j.jim.2006.12.010

[52] Ishii M, Kikuta J, Shimazu Y, Meier-Schellersheim M, Germain RN (2010) Chemorepulsion by blood S1P regulates osteoclast precursor mobilization and bone remodeling in vivo. The Journal of Experimental Medicine 207: 2793–2798. 21135136 10.1084/jem.20101474 PMC3005230

[53] Samadani A, Mettetal J, van Oudenaarden A (2006) Cellular asymmetry and individuality in directional sensing. Proc Natl Acad Sci U S A 103: 11549–54. 16864788 10.1073/pnas.0601909103 PMC1544207

[54] Swaney KF, Huang CH, Devreotes PN (2010) Eukaryotic chemotaxis: a network of signaling pathways controls motility, directional sensing, and polarity. Annu Rev Biophys 39: 265–89. 20192768 10.1146/annurev.biophys.093008.131228 PMC4364543

[55] Tang Y, Othmer HG (1994) A G protein-based model of adaptation in Dictyostelium discoideum. Math Biosci 120: 25–76. 8155908 10.1016/0025-5564(94)90037-x PMC6388623

[56] Dallon JC, Othmer HG (1997) A discrete cell model with adaptive signalling for aggregation of Dictyostelium discoideum. Philos Trans R Soc Lond B Biol Sci 352: 391–417. 9134569 10.1098/rstb.1997.0029 PMC1691935

[57] Szabo B, Szollosi GJ, Gonci B, Juranyi Z, Selmeczi D, et al (2006) Phase transition in the collective migration of tissue cells: experiment and model. Phys Rev E Stat Nonlin Soft Matter Phys 74: 061908. 17280097 10.1103/PhysRevE.74.061908

[58] Chate H, Ginelli F, Gregoire G, Peruani F, Raynaud F (2008) Modeling collective motion: variations on the Vicsek model. Eur Phys J B 64: 451–456.

[59] Dworkin M, Keller K (1977) Solubility and diffusion coefficient of adenosine 3′: 5′-monophosphate. Journal of Biological Chemistry 252: 864. 14137

[60] Song J, Guo LW, Muradov H, Artemyev NO, Ruoho AE, et al (2008) Intrinsically disordered gamma-subunit of cGMP phosphodiesterase encodes functionally relevant transient secondary and tertiary structure. Proc Natl Acad Sci U S A 105: 1505–10. 18230733 10.1073/pnas.0709558105 PMC2234174

[61] Vicsek T, Czirók A, Ben-Jacob E, Cohen I, Shochet O (1995) Novel Type of Phase Transition in a System of Self-Driven Particles. Phys Rev Lett 75: 1226–1229. 10060237 10.1103/PhysRevLett.75.1226

[62] Czirok A, Stanley H, Vicsek T (1997) Spontaneously ordered motion of self-propelled particles. J Phys A-Math Gen 30: 1375.

[63] Andrew N, Insall RH (2007) Chemotaxis in shallow gradients is mediated independently of PtdIns 3-kinase by biased choices between random protrusions. Nat Cell Biol 9: 193–200. 17220879 10.1038/ncb1536

[64] Das S, Rericha EC, Bagorda A, Parent CA (2011) Direct biochemical measurements of signal relay during Dictyostelium development. J Biol Chem 286: 38649–58. 21911494 10.1074/jbc.M111.284182 PMC3207423

[65] Rappel WJ, Nicol A, Sarkissian A, Levine H, Loomis WF (1999) Self-organized Vortex State in Two-Dimensional Dictyostelium Dynamics. Phys Rev Lett 83: 1247–1250.

[66] Liu X, Ma B, Malik AB, Tang H, Yang T, et al (2012) Bidirectional regulation of neutrophil migration by mitogen-activated protein kinases. Nat Immunol 13: 457–64. 22447027 10.1038/ni.2258 PMC3330201

[67] Golé L, Rivière C, Hayakawa Y, Rieu JP (2011) A quorum-sensing factor in vegetative Dictyostelium discoideum cells revealed by quantitative migration analysis. PLoS ONE 6: e26901. 22073217 10.1371/journal.pone.0026901 PMC3207821

[68] McLennan R, Dyson L, Prather KW, Morrison JA, Baker RE, et al (2012) Multiscale mechanisms of cell migration during development: theory and experiment. Development 139: 2935–44. 22764050 10.1242/dev.081471 PMC3403103

