# Supplementary figures and images for: Modeling and Measuring Signal Relay in Noisy Directed Migration of Cell Groups
Source: PLoS Comput Biol. 2013 May 2;9(5):e1003041. doi: 10.1371/journal.pcbi.1003041 (PMC3642071; doi:10.1371/journal.pcbi.1003041)

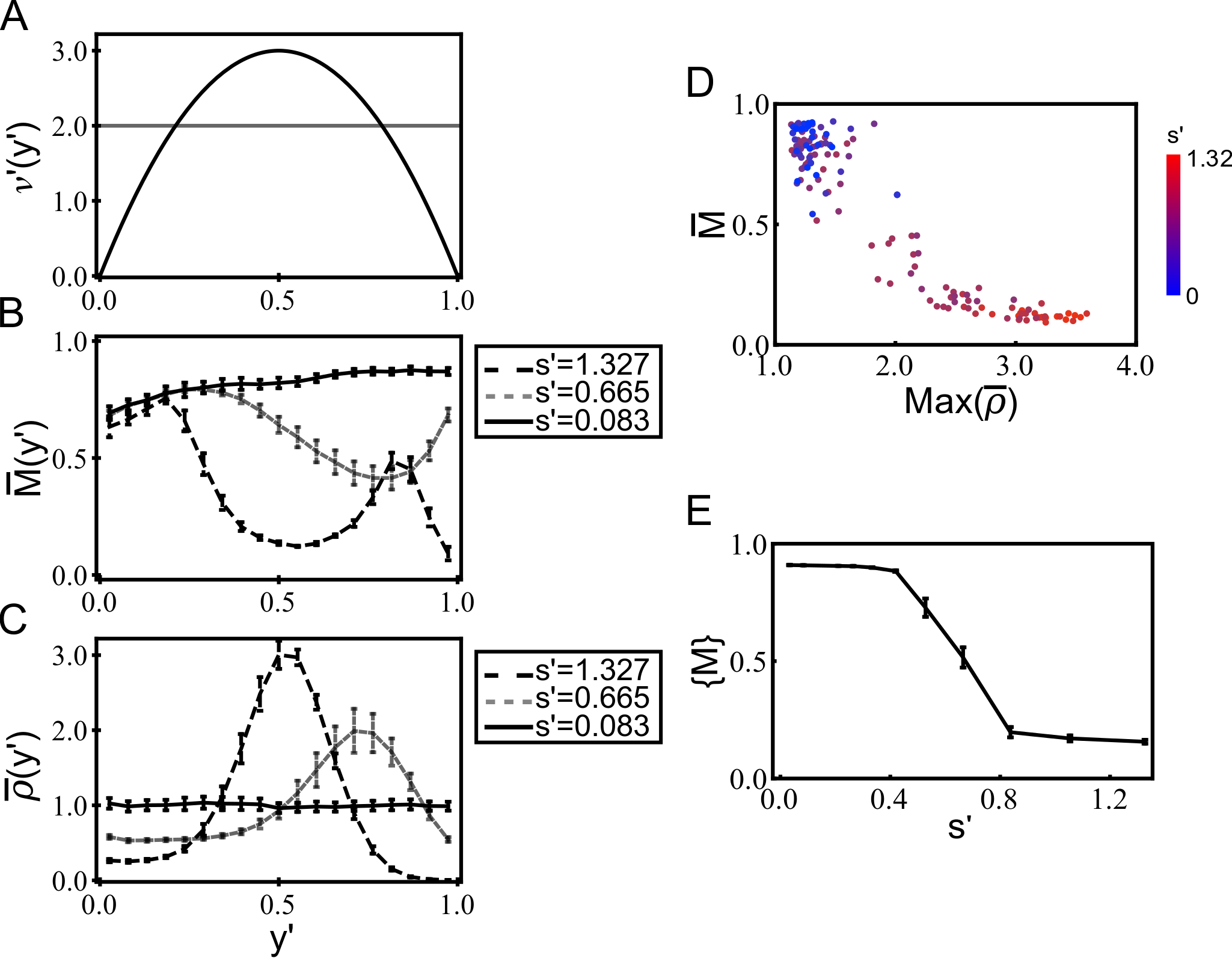

Supplement: Figure S1 — Results for the uniform cAMP degradation scheme. (A) The degradation rate as a function of the distance from the cell reservoir, where . (B) is shown for three representative relative cAMP secretion rates, whose dynamics is shown in Fig.3. (C) for the same relative cAMP secretion rates used in the upper panel. (D) Maximum in the region, is plotted against its corresponding for all numerical simulations with constant degradation scheme. Each point represents a single numerical realization and is color coded with respect to . (E) is plotted against , where the each data point is obtained from averaging many numerical realizations . The vertical bars represent the error in the mean, which is calculated by the standard error from many realizations. (TIF) [file pcbi.1003041.s001.tif]

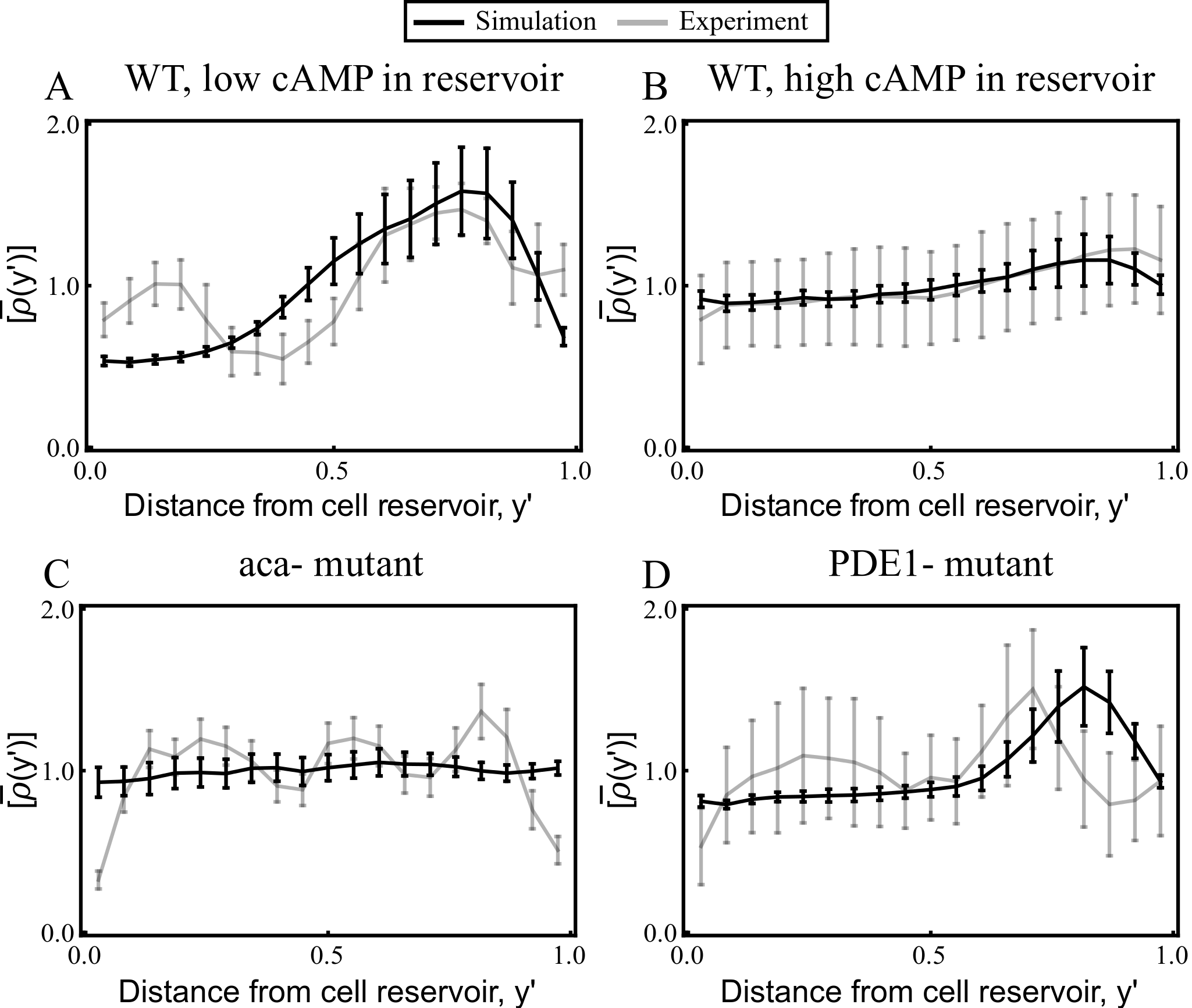

Supplement: Figure S2 — Density profile measurements. The density, , is plotted against the distance from the cell reservoir for wild-type cells moving in low cAMP concentration in the reservoir (left), wild-type cells moving in high cAMP concentration in the reservoir (center) and aca- mutant cells moving in high cAMP concentration in the reservoir (right). The density profile is obtained both from experiments and simulations of the model for (A) , , (B) , , (C) , , (D) , . Each simulation data point is obtained from averaging many numerical realizations. The vertical bars in both experimental and simulation data represent the standard error of the mean. (TIF) [file pcbi.1003041.s002.tif]
