# Supplementary material for: Modeling and Measuring Signal Relay in Noisy Directed Migration of Cell Groups
Source: PLoS Comput Biol. 2013 May 2;9(5):e1003041. doi: 10.1371/journal.pcbi.1003041 (PMC3642071; doi:10.1371/journal.pcbi.1003041)
Supplement: Text S1 — The supplementary text provides details regarding the assumptions used in our model in addition to comparison of numerical results with experimental observations. (PDF) [file pcbi.1003041.s003.pdf]

Supporting Information

Nonuniform hydrolysis profile approximation

To observe aggregation, cells are starved in a shaking flask with low concentration cAMP pulses for 5 hours. During this period they differentiate and secrete chemicals as a byproduct of the development process. Phosphodiesterase (PDE) that hydrolyzes the signaling molecule cAMP is secreted at a relatively constant rate during the preparation duration [1]. The activity of the PDE inhibitor is reduced for our method of pulsing [2]. Thus we approximate the PDE density as $n_{\mathrm{PDE}} = n_{\mathrm{cell}} s_{\mathrm{PDE}} T$, where n_{cell} is the cell density, s_{PDE} is the rate of PDE production per cell per unit time (assumed to be constant), and $T = 5$ hours is the total preparation time. Once the cells are placed in the $y < 0$ reservoir they settle quickly to its bottom surface. After settling is complete the cell surface density is $\bar{n}_{\mathrm{cell}} = n_{\mathrm{cell}} L_z$, where $L_z = 1$ cm is the height of the reservoir [3]. In the $0 \le y \le L_y$ region, the dynamics of the enzyme PDE reaches a steady state in about $(L_y/2)^2/D_{\mathrm{PDE}} \sim 4$ min. The $x - t$ average of the diffusion equation describing the dynamics of the PDE concentration $\bar{C}_{\mathrm{PDE}}$ is

$$D_{\mathrm{PDE}} \frac{d^2}{dy^2} \bar{C}_{\mathrm{PDE}} + s_{\mathrm{PDE}} \bar{n}_{\mathrm{cell}} = 0\,. \tag{SI1}$$

The vertical thickness of the chamber is very small $l_z = 5\mu$m. Therefore, within the chamber, the PDE concentration is considered uniform in this direction. As previously discussed, we assume the boundary conditions

$$\bar{C}_{\mathrm{PDE}}(0) = 0\,, \tag{SI2}$$

$$\bar{C}_{\mathrm{PDE}}(L_y) = 0\,. \tag{SI3}$$

The solution of Eq. (SI1) subject to the boundary conditions in Eqs. (SI2) and (SI3) is

$$\bar{C}_{\mathrm{PDE}}(y) = \frac{s_{\mathrm{PDE}} \bar{n}_{\mathrm{cell}}}{2D_{\mathrm{PDE}}} L_y^2 \frac{y}{L_y} \left(1 - \frac{y}{L_y}\right)\,. \tag{SI4}$$

Next, to justify (SI2) we argue that $\bar{C}_{\rm PDE}(L_y/2) \gg \bar{C}_{\rm PDE}(0)$ holds in our experimental setup. The PDE concentration at $y = 0$ is estimated by matching it to an estimate of the PDE concentration in the reservoir at $y < 0$, which is $n_{\rm cell} s_{\rm PDE} T$. Thus, Eq. (SI2) is valid if $(n_{\rm cell} s_{\rm PDE} T) l_z \ll \frac{s_{\rm PDE} \bar{n}_{\rm cell}}{8 D_{\rm PDE}} L_y^2$, where the factor l_z on the left hand side of this inequality results from the fact that $\bar{C}_{\rm PDE}$ is a surface density rather than a volume density. With $\bar{n}_{\rm cell} = n_{\rm cell} L_z$, the cell density and the unknown PDE production rate $s_{\rm PDE}$ cancel, and the inequality becomes

$$T \ll \frac{L_z}{l_z} \frac{L_y^2}{8 D_{\rm PDE}} \,. \tag{SI5}$$

Using the dimensions of the experimental setup and an estimate of the diffusivity of the PDE in Eq. (SI5), we obtain $T = 5$ hours $\ll \frac{1\text{mm}}{5\mu\text{m}} \frac{9 \times 10^4 \mu\text{m}^2}{8 \times 100 \mu\text{m}^2/\text{sec}} \approx 56$ hours. Therefore, the boundary condition assumed in Eq. (SI2) is reasonable. The other boundary condition Eq. (SI3) is even better justified because: (i) there are very much fewer cells in the $y > L_y$ reservoir than in the $y < 0$ reservoir, and (ii) the experiment time (≈ 1 hour) is shorter than T.

Fokker-Planck equation for aca- mutant cells

In this section we describe the steady state behavior of the model in the continuum approximation. For the non-interacting aca- cells, the cAMP density gradient always points toward the $y > L_y$ reservoir (i.e., $\nabla C / |\nabla C| = \hat{\mathbf{y}}$). Additionally, we set $\mathbf{f} \to 0$ for the continuum limit, thus the attractor vector in Eq. (2) reduces to $\mathbf{g} = \xi_x \hat{\mathbf{x}} + (1 + \xi_y) \hat{\mathbf{y}}$. Since $|\mathbf{n}| = 1$, therefore Eq. (2) reduces to

$$\frac{d\theta}{dt} = -\omega\theta + \omega\xi_x \,, \tag{SI6}$$

where θ is the angle between $\mathbf{n}$ and the y-axis. The steady state, spatially uniform version of the Fokker-Planck equation corresponding to (1) and (SI6) is

$$\frac{\partial}{\partial\theta} \left(\omega\theta\rho + \frac{\eta\omega^2}{2} \frac{\partial\rho}{\partial\theta} \right) = 0 \,, \tag{SI7}$$

where $\rho = \rho(x, y, \theta; t)$ is the probability density of the cells in (x, y, θ) space. Multiplying Eq. (SI7) by θ^2 and integrating over θ from $\theta = -\pi$ to $\theta = \pi$ with ρ assumed small away from $\theta \approx 0$ we obtain

$$\frac{\langle \theta^2 \rho \rangle}{\langle \rho \rangle} = \eta\omega/2\,. \tag{SI8}$$

Results for uniform degradation scheme

In our model we considered a non-uniform cAMP degradation scheme, which is justified by the boundary conditions of the setup and the initial conditions determined by the cell preparation. In this section we show results where we apply uniform cAMP degradation in the $0 < y' < 1$ region. The degradation for the external cAMP is treated spatially uniform in most of the other chemotaxis and collective cell migration models [4–7]. We summarize our results for the constant degradation scheme in Fig. S1, where we show $\overline{M}(y')$ and $\overline{\rho}(y')$ for $\nu_0 = 3$. Compared to the results obtained using the non-uniform cAMP degradation scheme (Figs. 4B, 4C, 4D, and 5A), results of the uniform cAMP degradation do not differ qualitatively.

Comparison of density profile measured from experiments

Tracking individual cells within a stream is technically difficult, however, since the depth of the experimental region is approximately the same as the thickness of the cell, we can infer local cell number from the images. More precisely, we thresholded and binarized the time-lapsed images to determine the z-projected area of the stream. We estimate this area is proportional to the number of cells within the stream. Figure S2 shows the local density obtained from experiments and simulations as a function of distance from the cell reservoir. Overall, both experiment with wild-type cells and simulation show an increase in density along the gradient direction and a peak density close to the high cAMP reservoir (Figs. S2A and S2B), with a stronger peak when the external cAMP concentration is low. The experiment and simulation disagree in the low external cAMP case near the cell reservoir. In the simulation, signal relay begins when the cells enter the thin gradient chamber. In the experiment, signal relay is not restricted and in low cAMP regions such as the cell influx well, the cells may begin to form streams. Mutant cells that do not secrete cAMP have a uniform density in the gradient chamber (Fig. S2C). To match the experimental density curve for the PDE1- cells, we lowered the cell secretion rate (Fig S2D). This result

suggests a testable prediction from our studies.

References

1. Gerisch G (1976) Extracellular cyclic-amp phosphodiesterase regulation in agar plate cultures of Dictyostelium discoideum. Cell Differ 5: 21–5.

2. Yeh RP, Chan FK, Coukell MB (1978) Independent regulation of the extracellular cyclic AMP phosphodiesterase-inhibitor system and membrane differentiation by exogenous cyclic AMP in Dictyostelium discoideum. Dev Biol 66: 361–74.

3. Kanegasaki S, Nomura Y, Nitta N, Akiyama S, Tamatani T, et al. (2003) A novel optical assay system for the quantitative measurement of chemotaxis. J Immunol Methods 282: 1–11.

4. Martiel JL, Goldbeter A (1987) A Model Based on Receptor Desensitization for Cyclic AMP Signaling in Dictyostelium Cells. Biophys J 52: 807–28.

5. Kessler DA, Levine (1993) Pattern formation in Dictyostelium via the dynamics of cooperative biological entities. Phys Rev E 48: 4801–4804.

6. Tang Y, Othmer HG (1994) A G protein-based model of adaptation in Dictyostelium discoideum. Math Biosci 120: 25–76.

7. Levine H, Aranson I, Tsimring L, Truong TV (1996) Positive genetic feedback governs cAMP spiral wave formation in Dictyostelium. Proc Natl Acad Sci U S A 93: 6382–6.
